# A low-carbohydrate diet may prevent end-stage renal failure in type 2 diabetes. A case report

**DOI:** 10.1186/1743-7075-3-23

**Published:** 2006-06-14

**Authors:** Jørgen Vesti Nielsen, Per Westerlund, Per Bygren

**Affiliations:** 1 Dept. of Medicine, Blekingesjukhuset, Karlshamn, Sweden; 2 Formerly Department of Nephrology, University Hospital, Lund, Sweden

## Abstract

An obese patient with type 2 diabetes whose diet was changed from the recommended high-carbohydrate, low-fat type to a low-carbohydrate diet showed a significant reduction in bodyweight, improved glycemic control and a reversal of a six year long decline of renal function. The reversal of the renal function was likely caused by both improved glycemic control and elimination of the patient's obesity.

Insulin treatment in type 2 diabetes patients usually leads to weight increase which may cause further injury to the kidney. Although other unknown metabolic mechanisms cannot be excluded, it is likely that the obesity caused by the combination of high-carbohydrate diet and insulin in this case contributed to the patient's deteriorating kidney function. In such patients, where control of bodyweight and hyperglycemia is vital, a trial with a low-carbohydrate diet may be appropriate to avoid the risk of adding obesity-associated renal failure to already failing kidneys.

## Background

In Sweden the number of patients with type 2 diabetes accompanied by end-stage renal failure has increased by 80 % since 1991 [1]. Control of blood glucose is crucial because of the proven link between HbA1c and the rate of decline of the kidney function in diabetic nephropathy [2]. Metabolic control in such patients is, however, difficult because the recommended low-fat diet with its high content of carbohydrates usually leads to a vicious cycle: hyperglycemia caused by the high-carbohydrate diet necessitates the use of insulin; efforts to normalise the blood glucose with insulin leads to increase of appetite and bodyweight [3]; the rise of bodyweight exposes the patient to the risk of obesity-associated renal failure [4]. A low-carbohydrate diet, however, is a potent antihyperglycemic remedy and may at the same time lead to weight loss [5,6].

We report here a significant reduction in body weight, improved glycemic control and reversal of a six year long decline of renal function in a patient with type 2 diabetes, whose diet we changed from the usually recommended high-carbohydrate, low-fat type to the opposite.

## Case report

The patient is a 60-year old man who was diagnosed with type 2 diabetes in 1989 and had a family history of overweight and diabetes. Retinopathy was diagnosed in the mid-90s. The patient had been treated several times with focal laser photocoagulation. In 2003 he developed proliferative retinopathy and was treated with photocoagulation. In addition to glimepiride and the ACE-inhibitor quinapril, medication included metoprolol, furosemide, simvastatin and aspirin. In 1995–1997, his blood pressure (BP) was on average 125/90 mm Hg. Over the same period, the patient's bodyweight varied between 89 kg and 85 kg. Traditional dietary counselling for weight loss usually resulted in a short-term loss of a few kg, but the bodyweight soon increased again.

In July 1997 albuminuria was noted with a urine concentration of 116 mg/l. That year the patient's average body mass index (BMI) was 29.4 kg/m^2 ^and the average bodyweight was 87.0 kg.

Insulin treatment was started four months later resulting in a temporary improvement of HbA1c but an increase in bodyweight. One year later, his BMI was 32.5 kg/m^2 ^and his weight was 94 kg. The insulin dose was lowered in order to avoid further increase in bodyweight. In the years 1998–1999, following the weight increase and despite improved glycemic control, his BP increased to an average 145/90 mm Hg. Likewise, urine albumin increased as seen in the Figure. In 2000, despite a still reasonable glycemic control but following further weight increase, the albuminuria reached an average of more than 2000 mg/l and the BP was 160/90 mm Hg. Both were controlled by exchanging quinapril for the angiotensin II receptor antagonist candesartan, and by adding amlopidine. The BP has since been stable, averaging 130/76 (± 10/7) mm Hg, but the decline of renal function continued. The Figure shows the albuminuria record and the increase in HbA1c, serum creatinine and body weight with increasing insulin dosage.

In January 2004 his diet was changed radically by reducing dietary carbohydrates to 80–90 g per day, consisting only of vegetables and small amounts of hard bread (crisp bread). Potatoes, bread, pasta, rice and cereals were excluded, and the caloric requirements were covered by protein and fat. To ease the transition the patient was supplied with a number of meal recipes suggesting a caloric restriction to about 1800 calories per day. The per cent proportions of carbohydrates, fat and protein in the recipes were 20 : 50: 30.

Less than two weeks later the patient discontinued his insulin treatment and 6 months later his bodyweight had decreased by 19 kg. HbA1c had dropped to 6.5 % after 3 months, and the steady rise of his serum creatinine stopped. The creatinine has since – for two-and-a-half years – been stable as seen in the figure. When insulin was discontinued rosiglitazone was prescribed. In the Table measured parameters are shown before the dietary change, and now. As of late 2005 there was no sign of proliferative disease in the patient's retinopathy.

## Discussion

As early as two years after the appearance of albuminuria, the patient had increased creatinine and urine albumin and higher BP. This happened despite a significantly improved glycemic control, although following an increase of bodyweight. A change of medication did affect BP and albuminuria but not the decline in kidney function. Only after a weight loss of 19 kg and an HbA1c reduction from a mean of about 8.5 % to 6.5 % was the steady decline of the kidney function reversed.

Diabetic nephropathy normally progresses to end-stage renal failure irrespective of the treatment. Only a complete 24 hour normoglycemia, as seen after pancreas transplants, can give the patient a chance to preserve the remaining kidney function.

In the Diabetes Control and Complication Trial (DCCT) a reduction of HbA1c from 9 % to 7 % (DCCT) led to a 40–50 % reduction in the number of patients whose kidney function deteriorated [2]. The reduction of HbA1c cannot by itself explain the change of the rate of decline. Also the HbA1c is far from normal. This level of hyperglycemia would not be able to produce a reversal to a completely preserved kidney function. Moreover, an almost normalised HbA1c in the years following the commencement of insulin treatment had no effect on the decline then.

## Obesity and renal failure

Obesity is a strong risk factor for renal failure especially in patients with diabetes and hypertension [4]. Even lean persons with central body fat distribution are at risk of having a lower rate of glomerular filtration [7]. An obesity-related glomerulopathy has been described [8]. The etiology may be ascribed to the fact that adipose tissue is a source of hormones including angiotensinogen, renin and leptin that may well influence renal function and BP [9,10]. Numerous inflammatory mediators such as TNFα, IL-6, resistin and others [11] are also secreted from fatty tissue. These contribute to chronic inflammation, general atherosclerosis and probably insulin resistance.

Beneficial effect of weight loss in proteinuric nephropathy has been shown in a controlled study [12]. A weight reduction of about 25 kg in an overweight diabetes patient after gastric by-pass lowered the proteinuria by 84 % and after further weight loss led to normalisation of a slightly elevated creatinine [13]. Finally, two case reports have described the partially resolving and stabilisation of dialysis-requiring renal failure after weight loss following bariatric surgery [14,15].

The common adverse effect of insulin treatment, the increase in bodyweight, may have contributed to the deterioration of the patient's kidney function in the years before the dietary change. Some degree of insulin sensitivity in the adipocytes is necessary for weight increase. During the first couple of years, with relatively modest insulin doses, the patient had a reasonable glycemic control while he increased in weight, which points at some degree of insulin sensitivity. By the year 2000, body weight may have exceeded an individual threshold for the accumulation of visceral fat after which insulin resistance increased [16 ] necessitating more insulin.

It may be assumed that improved glycemic control as well as weight loss contributed to stabilization of the patient's kidney function. The actual mechanisms behind the course of the patient's kidney disease are not known, however, and it cannot be excluded that other metabolic changes have contributed to the improvement of the renal function independently of weight loss. Even though other unknown metabolic pathways and mechanisms may have been involved, it is still unlikely that this patient would have avoided dialysis if his diet had not been changed. This is suggestive of a causal relationship between the diet and the course of the patient's kidney disease.

## Low-carbohydrate diet and weight loss

Low plasma insulin is a prerequisite for maximally stimulating the hormone-sensitive lipase that is responsible for lipolysis in fatty tissue. It therefore makes sense for an obese diabetes patient to reduce the high intake of carbohydrates. In this way the blood glucose is reduced and the patient is able to diminish or discontinue insulin. In addition, a low-carbohydrate diet with ad libitum food intake has been shown to be superior to the traditional calorie-restricted, low-fat, high-carbohydrate diet for weight loss in five randomised controlled studies [17-21]. The primary effect is caused by a lowering of appetite and a caloric intake reduced to the appropriate level for the patient's height [22].

The protein content of a diet may be a concern, but the actual size of the effect of protein restriction is modest [23,24]. It is also a misconception that a low-carbohydrate diet automatically is high in protein. This misconception may be a barrier for the use of a low-carbohydrate diet which is a highly effective tool in the management of type 2 diabetes. Such a diet can be modified so it suits the patient's needs i.e. the energy from carbohydrates can be replaced by energy from dietary fat and not necessarily from protein. The patient here was given a number of meal recipes at start. The aim was to give him the means to learn how to use this new dietary tool, which in essence leads to a completely different mind-set regarding diet. He was then recommended to consume about 70–90 g low-starch carbohydrates per day and to eat more fat. The patient is today still keeping the carbohydrates at the recommended level. He eats more fat and probably about 80–90 g of protein per day.

A motivating factor, in addition to being able to see that the kidney function has stabilized, was probably the increased feeling of well-being that followed very soon after the dietary change. Within 1–2 weeks of the dietary change – before any significant weight reduction had occurred – poor sleep and chronic fatigue was exchanged for a sound sleep pattern and increased vitality. This effect soon allowed the patient to again involve himself wholly in the running of his company. The reversal of the tiredness seen with chronic hyperglycemia may have been the cause. It may be that reversal of carbohydrate-induced memory impairment in type 2 diabetes played a role in restoring the patient's alertness and self-confidence [25 ].

## Conclusion

The present case report shows that a low-carbohydrate, high-fat diet improves glycemic control, reduces body weight and may prevent the development of end-stage renal failure in an overweight patient with type-2 diabetes. Furthermore, it raises the concern that the obesity caused by the combination of a high-carbohydrate diet and insulin may have contributed to the patient's failing kidney function.

## Competing interests

The author(s) declare that they have no competing interests.

## Authors' contributions

PB conceived the idea for the paper. JVN wrote the manuscript. PW and JVN analysed the data. All three gave final approval to the manuscript.

**Figure 1:**
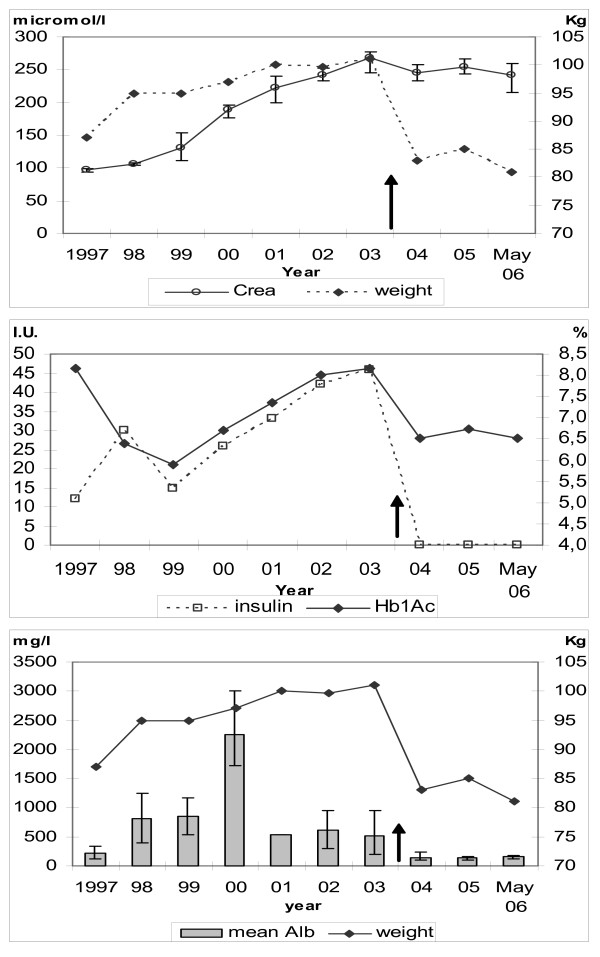
**Creatinine, bodyweight, HbA1c, insulin doses and urine-albumin**. The figures shown are the average per year (including the first 5 months of 2006) before and after a change to a low-carbohydrate diet in an obese patient with type 2 diabetes and renal failure. Arrow: Start of low-carbohydrate diet. Creatinine and urine albumin concentrations are given with ranges. Normal range: HbA1c <5.6 %. Creatinine 60–120 μmol/l. Urine albumin normal < 30 mg/l.

**Table 1:** The measured parameters before the dietary change, and current.

	**January 2004**	**May 2006**
**Weight (kg)**	102	81
**BMI (kg/m ^2 ^)**	34.5	27.4
**Blood pressure (mm Hg)**	135/75	130/75
**§HbA1c (%)**	9.4	6.5
**§Creatinine (μmol/l)**	269	215
**§BUN (mmol/l)**	15.3	14.4
**§Urine-albumin (mg/l)**	410	104
**Cholesterol (mmol/l)**	3.7	4.3
**HDL (mmol/l)**	0.8	1.3
**Triglycerides (mmol/l)**	1.8	2.1
**Ratio Chol/HDL**	4.6	3.3
**Ratio TG/HDL**	2.25	1.6

§ normal range: Creatinine = 60–120 μmmol/l; BUN = 3.2 – 8 mmol/l; urine-albumin <30 mg/l; HbA1c <5.6 %.
